# EWS‐FLI1 impairs aryl hydrocarbon receptor activation by blocking tryptophan breakdown via the kynurenine pathway

**DOI:** 10.1002/1873-3468.12243

**Published:** 2016-06-21

**Authors:** Cornelia N. Mutz, Raphaela Schwentner, Maximilian O. Kauer, Anna M. Katschnig, Florian Kromp, Dave N. T. Aryee, Sophie Erhardt, Michel Goiny, Javier Alonso, Dietmar Fuchs, Heinrich Kovar

**Affiliations:** ^1^ Children's Cancer Research InstituteSt. Anna KinderkrebsforschungViennaAustria; ^2^ Department of PediatricsMedical University ViennaAustria; ^3^ Department of Physiology and PharmacologyKarolinska Institutet StockholmSweden; ^4^ Unidad de Tumores Sólidos InfantilesInstituto de Investigación de Enfermedades Raras, ISCIII, CtraMadridSpain; ^5^ Division of Biological ChemistryBiocenter Innsbruck Medical University, Center for Chemistry and BiomedicineAustria

**Keywords:** aryl hydrocarbon receptor, EWS‐FLI1, tryptophan

## Abstract

Ewing sarcoma (ES) is an aggressive pediatric tumor driven by the fusion protein EWS‐FLI1. We report that EWS‐FLI1 suppresses TDO2‐mediated tryptophan (TRP) breakdown in ES cells. Gene expression and metabolite analyses reveal an EWS‐FLI1‐dependent regulation of TRP metabolism. TRP consumption increased in the absence of EWS‐FLI1, resulting in kynurenine and kynurenic acid accumulation, both aryl hydrocarbon receptor (AHR) ligands. Activated AHR binds to the promoter region of target genes. We demonstrate that EWS‐FLI1 knockdown results in AHR nuclear translocation and activation. Our data suggest that EWS‐FLI1 suppresses autocrine AHR signaling by inhibiting TDO2‐catalyzed TRP breakdown.

## Abbreviations

**3‐HK**, 3‐hydroxykynurenine

**AHR**, aryl hydrocarbon receptor

**ARNT**, AHR nuclear translocator

**bHLH‐PAS**, basic‐helix‐loop‐helix Per‐ARNT‐Sim

**DAPI**, 4′,6‐diamidino‐2‐phenylindole

**DRE**, dioxin response element

**dox**, doxycycline

**EF**, EWS‐FLI1

**EFH**, EWS‐FLI1‐high

**EF‐high**, EWS‐FLI1‐high

**EFL**, EWS‐FLI1‐low

**EF‐low**, EWS‐FLI1‐low

**ETS**, ETS transcription factor

**EWS**, Ewing sarcoma oncogene

**FICZ**, formylindolcarbazole

**FLI1**, Fli‐1 proto‐oncogene, ETS transcription factor

**IFN**, interferon

**IDO1**, indoleamine 2,3‐dioxygenase‐1

**KAT1**, kynurenine aminotransferase

**KMO**, kynurenine 3‐monooxygenase

**KYN**, kynurenine

**KYNA**, kynurenic acid

**LTR**, long terminal repeat

**MS**, mass spectrometry

**NAD^+^**, nicotinamide adenine dinucleotide

**RNAi**, RNA interference

**TDO2**, tryptophan 2,3‐dioxygenase‐2

**TRAIL**, TNF‐related apoptosis‐inducing ligand

**TRP**, tryptophan

**Figure 1:**
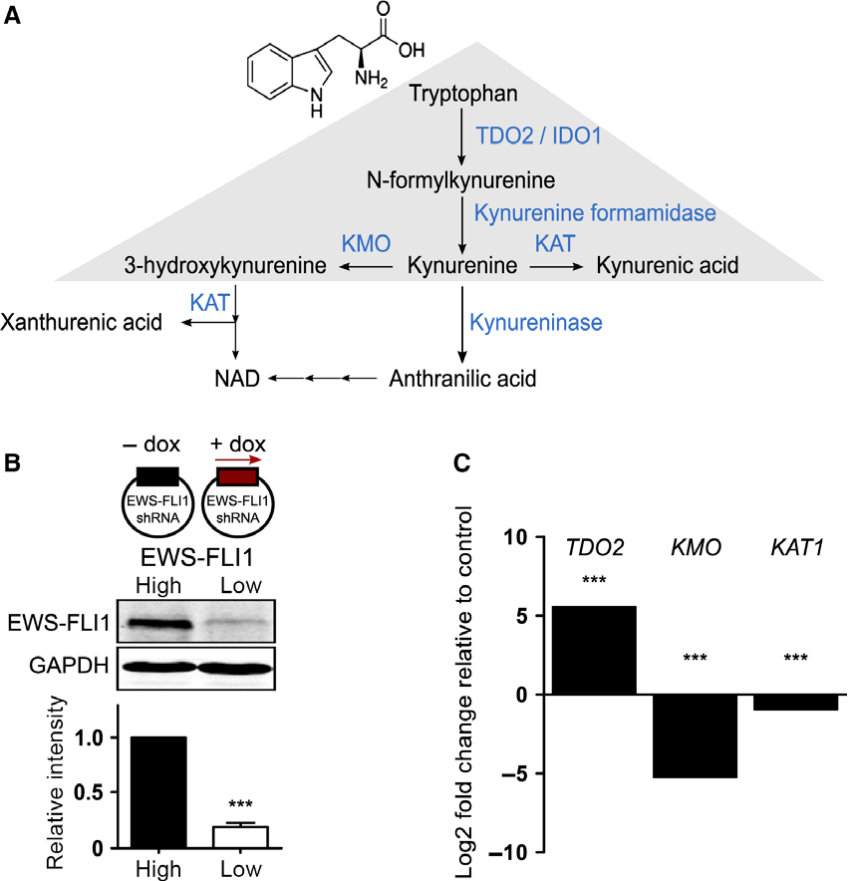
The KYN pathway of TRP metabolism. (A) Schematic diagram of the KYN pathway. TRP breakdown is initiated by TDO2 or IDO1 and the product is hydrolyzed to KYN. KYN itself can act as signaling molecule or can be the substrate for KMO, KAT, or kynureninase in order to fuel different pathways. Multiple arrows depict several enzymatic steps; gray triangle highlights KYN pathway. (B) A673sh cells harbor a dox‐inducible shRNA against EWS‐FLI1 allowing for switching from high (EWS‐FLI1‐high, EFH) to low (EWS‐FLI1‐low, EFL) EWS‐FLI1 expression levels. A673sh protein lysates were immunoblotted after 72 h induction of the shRNA. Western blot shows a representative experiment and quantification of protein expression ± SD from three replicates using LICOR Odyssey Infrared Imaging System is displayed in lower panel. ***P < 0.001. (C) Differential RNA expression of enzymes of the KYN pathway after EWS‐FLI1 depletion. *TDO2* expression increased and expression of *KMO* and *KAT1* decreased strongly. Values are shown as mean log2 fold change relative to EWS‐FLI1 expressing cells (data taken from 3). ***P‐value <0.001; Voom test statistic 71 adjusted for multiple testing by the Benjami Hochberg method.

Ewing sarcoma (ES) is the second most common primary malignant bone tumor in children and young adults. ES is characterized by the presence of a gene rearrangement between *EWSR1* and one of five different ETS transcription factor (ETS) genes, with *FLI1* being most commonly affected 1. The expression of EWS‐FLI1 (EF) results in the modulation of hundreds of different target genes 2. RNA‐sequencing data analysis revealed tryptophan 2,3‐dioxygenase‐2 (TDO2) as one of the genes being significantly up‐regulated after silencing EF in A673sh cells 3. We therefore investigated the involvement of EF in tryptophan (TRP) metabolism since little is known about the metabolic alterations caused by EF in cells, and also the repressive EF regulatory network is still poorly understood. Most of the dietary TRP, an essential amino acid, is metabolized along the kynurenine (KYN) pathway leading to the synthesis of NAD^+^ together with intermediate products, including KYN, 3‐hydroxykynurenine (3‐HK), and quinolinic acid 4. A secondary path from KYN leads to the generation of kynurenic acid (KYNA) via kynurenine aminotransferase‐1 (KAT1) 5. TDO2 and indoleamine 2,3‐dioxygenase‐1 (IDO1) are the first enzymes of the pathway (see Fig. 1A) with TDO2 being almost exclusively expressed in the liver and the brain 6, whereas IDO1 is found in tissues throughout the body 7. The KYN pathway has been implicated in a variety of diseases and disorders such as AIDS, Alzheimer's disease, depression, schizophrenia, Huntington's disease, amyotrophic lateral sclerosis, and neoplasia 8. Different metabolites of the KYN pathway have been associated with immune active properties 9, and because of their roles in immunity and the central nervous system, the KYN pathway has emerged as an attractive target for drug development 7. Several downstream metabolites of the KYN pathway are biologically active in various physiological and pathological processes, including KYN, KYNA, 3‐HK, anthranilic acid, 3‐hydroxyanthranilic acid, and quinolinic acid 10. KYNA has agonistic activity on the G protein‐coupled receptor GPR35 11 and antagonistic effects on glutamate receptors, in particular, the glycine co‐agonist site of the *N*‐methyl d‐aspartate (NMDA)‐receptor, and the cholinergic α7 nicotinic receptor, but it is also a ligand for the aryl hydrocarbon receptor (AHR) 12, 13. Similarly, KYN was identified as an endogenous AHR ligand in immune and tumor cells, acting both in an autocrine and paracrine manner, and promoting tumor cell survival 14. AHR belongs to the subgroup of basic‐helix‐loop‐helix Per‐ARNT‐Sim (bHLH‐PAS) transcription factors 15, best known as a receptor for xenobiotics such as polycyclic aromatic hydrocarbons 16. Ligand activation of AHR leads to its translocation to the nucleus, dimerization with AHR nuclear translocator (ARNT) 17, and binding to dioxin response elements (DRE) in the promoter region of target genes. Among these targets are genes encoding enzymes for xenobiotic metabolism, such as the cytochromes P450 CYP1A1/2 and CYP1B1, several phase II conjugating enzymes 15, 18, and pro‐inflammatory interleukins (IL)‐8 14, IL‐1β, and IL‐6 19. Analysis of large‐scale gene expression, chromosomal copy number, and massively parallel sequencing data of 947 human cancer cell lines from the Cancer Cell Line Encyclopedia identified elevated AHR as mechanistic biomarker for enhanced MEK inhibitor sensitivity in NRAS‐mutant cell lines 20. In addition, exposure to toxic polycyclic aromatic hydrocarbons stimulating the AHR has been implicated in a variety of cancers in experimental animals and humans 21.

The majority of genes activated by EF annotates to cell‐cycle regulation and proliferation, in contrast to genes down‐regulated by EF that mainly associate with cell differentiation and cell communication 22. Our study was performed in A673sh cells 23, where EF can be silenced via doxycycline (dox)‐inducible RNA interference (RNAi). Here, we identify a new signaling pathway that is activated when EF expression is low. It is induced via TRP breakdown and accumulation of intermediate metabolites in A673sh cells, which might play an important role in the pathogenesis of ES. This is the first report, to our knowledge, investigating the regulatory function of EF on the KYN pathway.

## Materials and methods

### Materials

The TDO2‐shRNA expression cassettes consist of a 29 nucleotide (nt) target‐gene‐specific sequence, a 7‐nt loop, and another 29 nt reverse complementary sequence, all under the control of a human U6 promoter. Constructs against human TDO2 (#1, 5′‐GGAGACGATGACAGCCTTGGACTTCAATG‐3′; #2, 5′‐CGGTGGTTCCTCAGGCTATCACTACCTGC‐3′) were purchased from Origene Technologies (Rockville, MD, USA). Additionally, the expression vector contains both 5′ and 3′ long terminal repeats (LTR) of Moloney murine leukemia virus (MMLV) that flank the puromycin marker and the U6‐shRNA expression cassette. KYN, KYNA, and the AHR inhibitor CH‐223191 were from Sigma‐Aldrich (St. Louis, MO, USA).

### Cells

A stably transfected subclone of A673 cells (A673sh) with a dox‐inducible shRNA against the EF fusion protein was used 23. Cells were kept in DMEM + GlutaMax supplemented with 10% fetal calf serum (FCS), 100 U·mL^−1^ penicillin, and 100 μg·mL^−1^ streptomycin (Gibco by Life Technologies, Carlsbad, CA, USA), 50 μg·mL^−1^ zeocin, and 2 μg·mL^−1^ blasticidin (InvivoGen, San Diego, CA, USA). In order to induce the EF shRNA, 1 μg·mL^−1^ dox (Sigma‐Aldrich) was added to the medium. Cells were transfected using the Lipofectamine Plus reagent (Invitrogen, Groningen, NL, USA) and on the following day, selection for efficiently transfected cells was performed with puromycin (InvivoGen). Preparation of fractionated cell extracts was accomplished using the NE‐PER Nuclear and Cytoplasmic Extraction Reagents Kit according to the manufacturer's instructions (Thermo Fisher Scientific, Waltham, MA, USA).

### Analysis of tryptophan metabolites

A673sh cells were treated with 1 μg·mL^−1^ dox for 48 and 72 h and kept in DMEM + 10% FCS throughout cultivation. Full medium used contained 83.5 ± 4.3 μm TRP, 0.6 ± 0.1 μm KYN, 39.3 ± 7.1 nm KYNA, and 2.3 ± 2.0 nm 3‐HK. Cell culture supernatant was collected, centrifuged, and transferred to fresh tubes and frozen until subjected to analysis.

TRP and KYN were measured as described previously 24. In brief, their concentrations were determined by HPLC on reversed‐phase C18 columns and subsequent monitoring of their UV absorption at 360 nm (KYN) and fluorescence (TRP) at 286 nm excitation and 366 nm emission wavelengths. For the analysis of KYNA 20 μL of each cell culture supernatant was injected on an HPLC column (Reprosil 100 C18, 3 μm particles, 100 × 4 mm, Dr. Maisch HPLC GmbH, Ammerbuch, Germany). The analytes were eluted with an acetonitrile/sodium acetate (6.8%/30 nm) solution at a flow rate of 0.5 mL·min^−1^. The eluate was then mixed on line with zinc acetate (final concentration 0.125 m) and KYNA was determined with a fluorescence detector set at excitation and emission wavelengths of 344 and 398 nm, respectively. The signals from the fluorescence and UV‐VIS detectors were transferred to a computer and analyzed by datalys azur software (Grenoble, France). The concentration of KYNA was extrapolated from freshly prepared standard curves. To determine 3‐HK concentration, 20 μL was subjected to analysis utilizing an isocratic reversed‐phase HPLC system coupled to an electrochemical detector (Coulochem III; ESA Inc.) with an applied potential of 100–150 mV. A mobile phase consisting of 20 mm sodium phosphate, 0.7 mm octane sulfonic acid, and 8% acetonitrile (pH 3.2) was pumped through a Reprosil‐Pur C18 column (4 × 150 mm; Dr. Maisch HPLC GmbH), at a flow rate of 0.6 mL·min^−1^. Signals from the detector were analyzed using the clarity software (Data Apex Ltd, Prague, Czech Republic). The concentrations were calculated based on standard solutions.

### Luciferase reporter assay

The wild‐type pT81/3xDRE and mutant pT81/CDEF plasmids were a kind gift from Peter A. Münzel (Department of Toxicology, University of Tübingen, Germany). The pT81/3xDRE reporter construct was generated using the motif of DRE3 from the CYP enhancer region fused in triplicate tandem array into pT81Luc as previously described 25. Cells were cotransfected with the pT81‐based reporter constructs and pRL‐TK (Promega, Madison, WI, USA) using Lipofectamine Plus reagent (Invitrogen) at 20% density. The cells were treated with dox, KYN (50 μm), KYNA (150 nm), or AHR inhibitor (6 μm; CH‐223191) 24 h after transfection, and gene reporter assays were carried out with the Dual Glo Luciferase assay kit (Promega) 96 h after transfection (48 h compound/dox treatment). Renilla Luciferase activity served as a measure of transfection efficiency.

### Immunoblot analysis

Total proteins were resolved by 6.5% or 8.5% SDS/polyacrylamide gel electrophoresis and processed for immunoblotting. Antibodies were: AHR (Cell Signaling Technology, New England Biolabs GmbH, Frankfurt, Germany), FLI1 (MyBiosource, San Diego, CA, USA), Lamin A/C (Santa Cruz, Dallas, TX, USA), TDO2 (Abcam, Cambridge, UK), Vinculin (Sigma, Darmstadt, Germany), α‐Tubulin (Calbiochem, San Diego, CA, USA). Preparation of fractionated cell extracts was accomplished with NE‐PER Nuclear and Cytoplasmic Extraction Reagents (Thermo Fisher Scientific). Blot detection was performed with the LI‐COR Odyssey Infrared Imaging System (LI‐COR Biosciences, Lincoln, NE, USA).

### Immunofluorescence microscopy

Cells were fixed with 4% paraformaldehyde in PBS for 10 min at room temperature. Cells were permeabilized with 0.3% Triton^™^ X‐100 in PBS with 5% goat serum (Dako, Agilent Technologies, Santa Clara, CA, USA) for 30 min. Subsequently, the primary rabbit anti‐AHR antibody (Abcam) was added in 0.1% Triton^™^ X‐100/1% bovine serum albumin (BSA)‐PBS with 1% goat serum overnight at 4 °C. The secondary goat anti‐rabbit Alexa Fluor 488 antibody (Life Technologies) was diluted in 0.1% Triton^™^ X‐100 in 2% BSA‐PBS with 1% goat serum and added for 30 min at room temperature. Cells were mounted with Vectashield mounting medium containing 4′,6‐diamidino‐2‐phenylindole (DAPI) (Vector Laboratories, Burlingame, CA, USA). Immunostainings were visualized at 63× magnification using the Leica TCS SP8 confocal microscope and images were taken using the Leica las‐af software (Leica Microsystems, Wetzlar, Germany). For analysis of acquired images at single‐cell level, Matlab‐based statistical methods were applied. This approach allowed for detection of nuclei with DAPI/AHR stained images in order to determine morphological parameters and marker intensities. All of the features were used to create image scatter plots and hierarchical gating strategies were applied to correct for imaging artifacts 26, 27.

### RNA preparation and reverse transcription‐quantitative PCR (RT‐qPCR)

Total RNA was prepared using the RNAeasy kit (Qiagen, Hilden, Germany). Transcripts were quantified by reverse transcription PCR using the ABI Prism 7500 Detection System (Applied Biosystems, Foster City, CA, USA). Real‐time PCR was performed with Maxima SYBR Green/ROX qPCR Master Mix (2×) (Thermo Scientific, Waltham, MA, USA). The relative quantification in gene expression was calculated with the use of the 2^−ΔΔ*C*t^ method 28. This method reveals the fold changes in gene expression normalized to an internal control gene (*GAPDH*). Primers used were the following: *TDO2* 5′‐ACTCCCCGTAGAAGGCAGCGAA‐3′ and 5′‐ CGGTGCATCCGAGAAACAACCT‐3′; GAPDH 5′‐TTCACCACCATGGAGAAGGC‐3′ and 5′‐GGAGGCATTGCTGATGATCTTG‐3′; *EWS‐FLI1* 5′‐ACTCCCCGTTGGTCCCCTCC‐3′ and 5′‐TCCTACAGCCAAGCTCCAAGTC‐3′; *IL8* 5′‐ATGACTTCCAAGCTGGCCGT‐3′ and 5′‐TCTCAGCCCTCTTCAAAAAC‐3′; *IL1B* 5′‐CTCGCCAGTGAAATGATGGCT‐3′ and 5′‐GTCGGAGATTCGTAGCTGGAT‐3′; *IL6* 5′‐ AGCCACTCACCTCTTCAGAACGAA‐3′ and 5′‐ AGTGCCTCTTTGCTGCTTTCACAC‐3′; *FAM65B* 5′‐GAAAGGCGATCCAGGTGTA‐3′ and 5′‐CCCTTTTAGGCTGAGGCTCT‐3′; *TUFT1* 5′‐GGAAAGTCCGGCAAATGATA‐3′ and 5′‐GCTGAAGTTGCCATGACTGA‐3′.

### Statistical analysis

Results are shown as representative images or as means ± SD of at least three independent experiments. If not stated otherwise, data were analyzed using the unpaired *t*‐test with Welch's correction or with the one‐sample *t*‐test using the prism 5 for Windows (version 5.02) statistical software (GraphPad Prism Software Inc., La Jolla, CA, USA). Data shown in graphical format represent the means (±SD) and a *P*‐value of ≤ 0.05 is considered statistically significant.

## Results

### EWS‐FLI1 knockdown activates tryptophan metabolism via the KYN pathway

**Figure 2:**
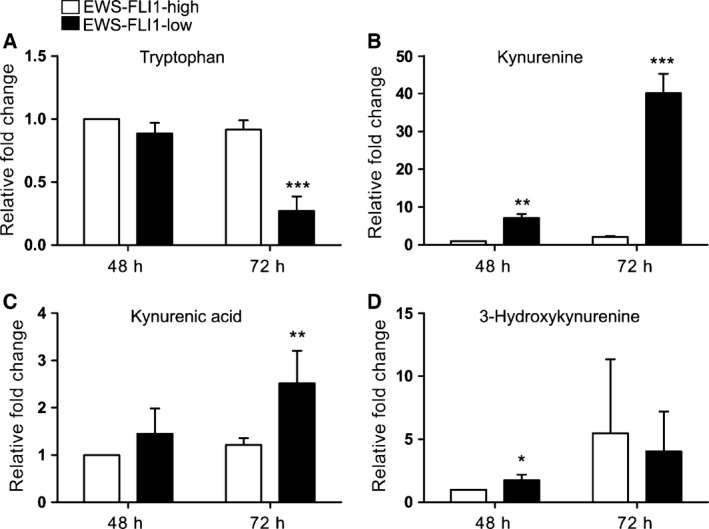
EWS‐FLI1 knockdown initiates TRP breakdown and leads to the accumulation of metabolites of the KYN pathway. A673sh cells were grown (EWS‐FLI1‐high, EFH) or treated with dox (EWS‐FLI1‐low, EFL) for 48 or 72 h and cell culture supernatants were analyzed. Prior experiment start, fresh full medium + 10% FCS was added and kept on the cells until collection. Results were normalized to control medium without cells and data are shown as relative fold change over control conditions for EFH cells (white bars) and EFL cells (black bars). (A) TRP decreased in cells where EWS‐FLI1 has been silenced for 72 h, whereas the downstream metabolites of TRP, KYN, and KYNA, were elevated (B, C). (D) 3‐HK increased slightly faster in the supernatant of EFL than of EFH cells at 48 h of incubation, while no difference in 3‐HK accumulation was detected after 72 h. Data are shown as means ± SD from four independent experiments. *P < 0.05, **P < 0.01, ***P < 0.001.

The addition of dox to A673sh cells induces a knockdown of EF (Fig. 1B). Various enzymes of TRP metabolism, such as *TDO2*, kynurenine 3‐monooxygenase *(KMO*), *KAT1* are modulated after dox‐induced EF silencing for 53 h in A673sh cells (Fig. 1C 3). Therefore, we interrogated the pathway by measuring up‐ and downstream metabolites of KYN (Fig. 2), a readout metabolite of TDO2 activity. Cells were incubated with fresh growth medium (including antibiotics and 10% FCS as described in ‘Materials and Methods’) in absence or presence of dox for 48 and 72 h and media from EWS‐FLI1‐high (EFH) and EWS‐FLI1‐low (EFL) cells were collected and subjected to HPLC and mass spectrometry (MS) analyses for the metabolites TRP, KYN, KYNA, and 3‐HK. In EFL cell supernatants, TRP levels were significantly reduced after 72 h (Fig. 2A). KYN was hardly detectable in the media of EFH cells, but was highly enriched (up to 40‐fold) upon EF depletion (Fig. 2B). Although EFH cells produced small amounts of the downstream metabolite KYNA, the levels were highly elevated after EF knockdown, most prominently at 72 h of dox treatment (Fig. 2C). KYNA is known to inhibit the proliferation of various cancer cell lines 29, 30 and also acts as a neuroprotective agent in the central nervous system 31. Up to 72 h of incubation, 3‐HK increased in the supernatants of A673sh cells under both conditions, but slightly faster upon EF silencing (Fig. 2D). The metabolite measurements suggest that in the absence of EF, TRP breakdown was initiated and led to the accumulation of KYN and KYNA, which are both known AHR ligands 12, 32.

### TDO2 is responsible for tryptophan uptake in EWS‐FLI1‐low cells

**Figure 3:**
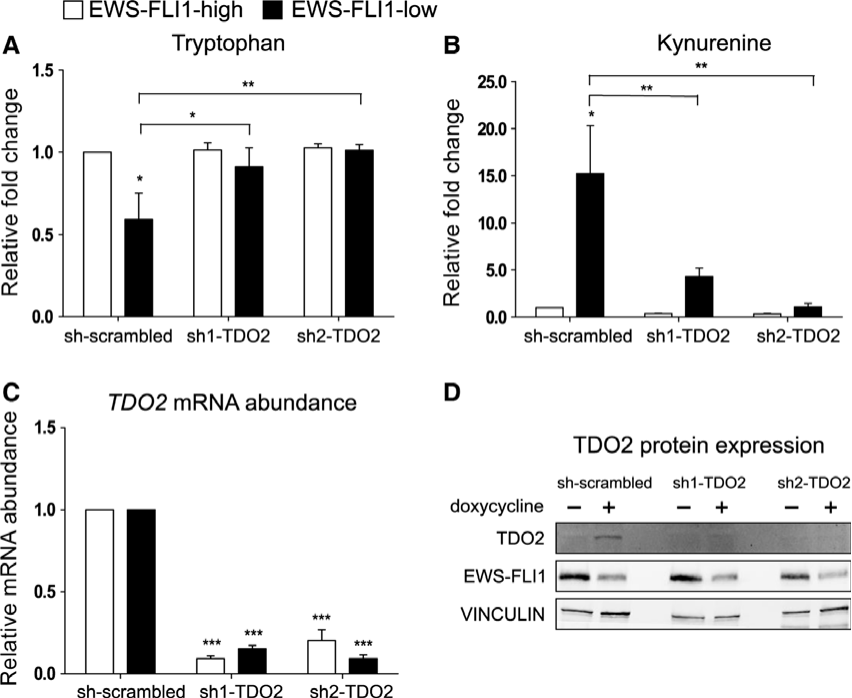
Silencing of TDO2 upon EWS‐FLI1 knockdown inhibits TRP consumption and KYN accumulation. White bars indicate EFH, black bars EFL conditions. Two different shRNA constructs (sh1‐ and sh2‐TDO2) against *TDO2* and a scrambled shRNA control were used for transfecting A673sh cells. On the following day, cells were selected with fresh medium + puromycin with or without the addition of dox for a total of 72 h. (A, B) Cell culture supernatants were collected for HPLC‐MS analysis of TRP and KYN. Both shRNAs—applied individually—reversed the TRP consumption and KYN accumulation compared to scrambled shRNA. Results are shown normalized to control medium and displayed as relative fold change. (C) *TDO2* mRNA was analyzed with quantitative real‐time PCR (qRT‐PCR). *GAPDH* served as housekeeping gene. Both shRNA constructs (sh1‐, sh2‐TDO2) lead to a decrease of *TDO2* mRNA abundance after 72 h of puromycin selection. Data were normalized to sh‐scrambled of EFH condition. Data are shown as means ± SD from four independent experiments. *P < 0.05, **P < 0.01, ***P < 0.001. (D) Representative immunoblot analysis for TDO2 expression after transfection with sh‐scrambled, sh1‐TDO2, and sh2‐TDO2 upon EWS‐FLI1‐high and EWS‐FLI1‐low conditions. Transfected cells were selected with puromycin for 72 h and were treated with dox (72 h) in parallel. Vinculin served as loading control.

In order to verify whether TDO2 is required for TRP uptake in EF silenced A673sh cells, we investigated TRP consumption after silencing *TDO2* with two distinct shRNA constructs (sh1‐TDO2, sh2‐TDO2). One day post transfection, fresh full medium with puromycin (for selection) and with/without dox (for EF silencing) was added to the cells and kept throughout 72 h. Subsequently, medium was tested for TRP and KYN abundance (Fig. 3A,B). Both constructs led to a similar reduction in *TDO2* mRNA and protein levels of 70–90% (Fig. 3C,D). Cells harboring the sh‐scrambled control shRNA showed a strong decrease in TRP levels upon EF knockdown. However, in sh‐TDO2 transfected cells, TRP levels in the supernatant of EFL cells remained comparable to those of EFH cells (Fig. 3A), indicating that TDO2 activity is the main cause for TRP consumption after EF knockdown. Similarly, knockdown of *TDO2* prohibited KYN accumulation associated with EF silencing (Fig. 3B). As IDO1 is not expressed in EFH or EFL A673sh cells (Fig. S1), these data confirm that KYN levels are regulated by TDO2 in A673sh cells in the absence of EF.

### Functional activation of AHR is regulated by EWS‐FLI1

**Figure 4:**
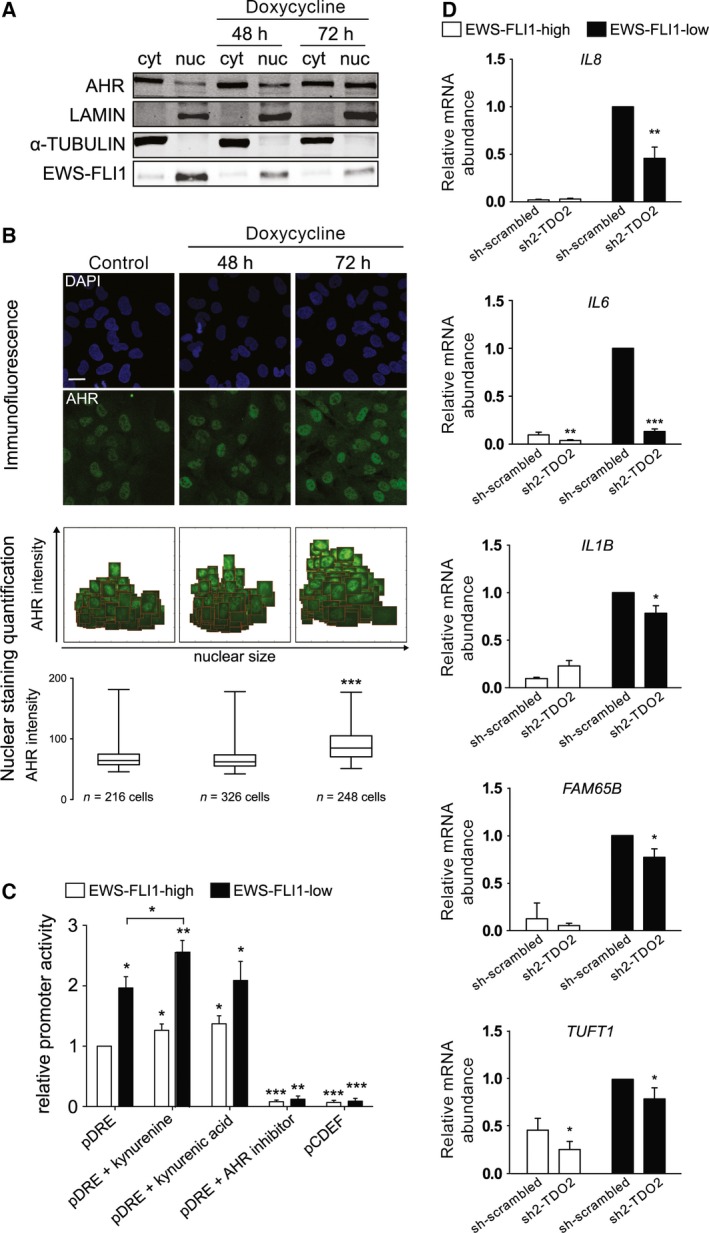
Subcellular localization, DRE binding of AHR, and AHR‐target genes are regulated by EWS‐FLI1. (A) Immunoblot analysis of endogenous AHR expression after separation of cytoplasmic (cyt) from nuclear (nuc) fractions. A673sh cells were treated with dox for either 48 or 72 h and subjected to cellular fractionation followed by western blotting for AHR, LAMIN (nuclear loading control), α‐TUBULIN (cytoplasmic loading control), and EWS‐FLI1. Lanes 1–2 illustrate EFH, lane 3–6 EFL cells. Data shown are a representative of three replicates. (B) Representative fluorescence microscopy images (upper panel) for AHR subcellular localization in the presence or absence of EWS‐FLI1. Endogenous AHR protein was monitored 48 and 72 h post EWS‐FLI1 depletion by dox treatment, using the anti‐AHR antibody (green) with DAPI counterstaining to delineate cell nuclei (blue). In the presence of EWS‐FLI1, AHR expression was negligible (control). After 48 and 72 h of dox treatment the AHR staining was confined almost exclusively to the nuclei. Scale bar = 25 μm. In the middle panel, representative nuclear staining quantifications are shown as scatter plots with clipped nuclei of AHR (green) and displayed as box plots in the lower panel. Mean intensities for nuclear AHR signals of 216 control cells were compared to those of 326 cells of 48 h and 248 cells of 72 h dox treatment. Statistics were performed using One‐Way ANOVA followed by Bonferroni post‐testing. *P < 0.05, **P < 0.01, ***P < 0.001. (C) Promoter activity of the wild‐type pT81/3xDRE (pDRE) plasmid carrying the DRE and DRE‐mutant pT82/CDEF (pCDEF) in EFH (white) and EFL (black) cells with shRNA‐EWS‐FLI1 induction for 48 h. Additionally, cells were treated with KYN (50 μm), KYNA (150 nm), or the specific AHR antagonist CH‐223191 (6 μm) for 48 h. Promoter activity was higher in EFL cells. Data shown are the means of three independent experiments ± SD, normalized to Renilla luciferase. *P < 0.05, **P < 0.01, ***P < 0.001. (D) mRNA expression of AHR target genes *IL8*,*IL1B*,*IL6*,*FAM65B*, and *TUFT1* in sh‐scrambled and sh2‐TDO2 conditions. *GAPDH* served as internal control gene. For facilitating visualization of AHR target genes, these data were normalized to sh‐scrambled of EFL cells. Data shown represent four independent experiments ± SD. *P < 0.05, **P < 0.01, ***P < 0.001.

Since we have demonstrated that KYN and KYNA are significantly up‐regulated in the culture medium of EF silenced A673sh cells, we wanted to elucidate functional consequences of these enriched metabolites. As KYN and KYNA are both ligands of the AHR 12, 32, we speculated that ligand activation of AHR signaling might be efficiently suppressed in the presence of EF and only initiated in its absence. In an inactive state the AHR rests in a cytosolic multiprotein complex including the heat shock protein 90 33. Upon activation, AHR translocates into the nucleus and enforces transcription via binding to DREs in the promoter region of target genes 34. This is accomplished in a dimeric form with ARNT 35. Thus, the subcellular localization of the AHR transcription factor depends on ligand binding. To investigate whether AHR is present in the nucleus of EFL cells, we separated the cytoplasmic from the nuclear cellular fractions and immunoblotted them with anti‐AHR antibody to visualize endogenous AHR expression (Fig. 4A). Nuclear localization of EF and its depletion upon dox treatment served as control. AHR protein was found in the cytoplasm and, to a much lesser extent, in the nucleus of EFH cells (Fig. 4A). However, upon EF silencing, AHR strongly increased in the nuclear fraction with the highest level of induction at 72 h of EF depletion. This finding is strong evidence for successful AHR activation and subsequent translocation to the nucleus in EFL cells. In concordance with western Blot analysis, immunofluorescence microscopy including quantification of nuclear AHR signal intensity supported these results (Fig. 4B). Although already present in the nucleus in EFH cells, a strong increase in nuclear AHR staining was observed after dox treatment. To further confirm the influence of EF on the activation of AHR, we performed luciferase reporter assays. After functional stimulation of DREs on the reporter vector, luciferase activity can serve as readout (Fig. 4C). EFL cells displayed significantly elevated luciferase activity which was lost in the presence of AHR inhibitor CH‐223191. Consistent with this finding, mutation of the DRE in pCDEF abolished reporter activity under EFL conditions (Fig. 4C). To follow up on the hypothesis that AHR gets activated after EF knockdown due to higher KYN and KYNA levels, the metabolites were added under EFH and EFL conditions. The concentrations of KYN and KYNA in the experiments were chosen according to their abundance in EFL conditions from MS analysis. Addition of KYN and KYNA led to increased DRE reporter activity.

Consistent with the reporter assay results, EF silencing drastically up‐regulated endogenous AHR‐target genes *IL8*,* IL6*,* IL1B*,* FAM65B*,* TUFT1, CYP1B1,* and *CYP1A1*. Knocking down *TDO2* in EFL conditions significantly decreased this up‐regulation in AHR‐target mRNA expression for most of the genes (*IL8*,* P* = 0.0027; *IL6*,* P* = 0.0001; *IL1B*,* P* = 0.012; *FAM65B, P* = 0.014; and *TUFT1*,* P* = 0.039) compared to sh‐scrambled EFL (Fig. 4D), but not for *CYP1B1* or *CYP1A1* (not shown). Collectively, these data suggest that EF represses AHR activity in A673sh cells. The receptor gets activated once EF is down‐regulated, which is most probably accomplished via initiation of TRP breakdown followed by an enrichment of KYN and KYNA.

## Discussion

With the advent of sensitive single‐cell gene expression analysis methods, it is becoming increasingly clear that stochastic variation in oncogene expression levels exist between individual cells of a tumor. This was recently demonstrated for FUS‐DDIT3 positive myxoid liposarcoma 36 and discussed for EF in ES at the 2nd European Ewing Sarcoma Research Summit 37. Here, such stochastic variations in EF expression may translate into differential metastatic behavior, since EF‐low cells were demonstrated to have drastically increased migratory and metastatic potential 38. In this study, we have used RNAi induced EF silencing as an experimental approach to study the phenotype of A673sh cells under EF‐low conditions. We report activation of TDO2 resulting in TRP degradation and KYN/KYNA dependent activation of AHR signaling and speculate that TRP degradation, KYN/KYNA accumulation and AHR activation contribute to the survival of A673sh cells in an autocrine manner under conditions when EF expression is low.

TRP degradation in cancers has mostly been attributed to the activity of IDO1 in cancer cells and tumor‐draining lymph nodes 39. Alternatively, in the absence of IDO1, TDO2 can overtake the constitutive TRP breakdown and KYN production in some human cancers and specialized myeloid cells 14. Several reports point out that IDO1 activity and IDO1 pathway play key roles in regulating immune evasion by tumors 40. IDO1 is expressed in several human cancers such as malignant melanoma, ovarian cancer, and colorectal cancer 41, 42. In the tumor microenvironment, IDO1‐mediated TRP deficiency leads to the induction of a stress response which finally results in cell‐cycle arrest of T‐cells 43, differentiation of T regulatory cells and an immunosuppressive environment 44. Additionally, the TRP degradation product KYN stimulates tumor promoting immune tolerance via activation of the AHR pathway 40, 45. Thus, IDO1 inhibition seems a promising strategy for cancer treatment and some antitumor effects have already been reported for human endometrial carcinoma and murine glioma 46, 47, 48.

However, IDO1 is not expressed in various tested ES cell lines (including A673) independently of EF levels, unless interferon (IFN) γ treated (Fig. S1) 49. Instead, we find TDO2 as the exclusive TRP degrading enzyme expressed in A673sh cells under EF‐low conditions resulting in the activation of AHR signaling. Both, AHR and ARNT are expressed in EFH and EFL cells 3, but nuclear localization of AHR was predominantly observed under EFL conditions (Fig. 4B,C). Expression of *TDO2* as well as genes with AHR binding sites such as *IL8*,* IL1B*,* IL6*,* FAM65B*,* TUFT1*,* CYP1B1,* and *CYP1A1* 3, 50 were low in the presence of EF *in vitro* (A673sh), but up‐regulated after EF silencing 3. Interestingly, CYP1A1 is predominantly involved in detoxification, whereas CYP1B1 is required for metabolic activation in favor of tumor initiation 51. In the AHR expressing state of EFL cells, *CYP1B1* is expressed to a very high extent compared to *CYP1A1* 3, 50, probably a result of metabolic activation and proliferation. This speculation might be counterintuitive considering the fact that depleting ES cells of EF has been associated with growth inhibition and G1 cell‐cycle arrest 52, 53. However, although a large proportion of EFL cells enters cell‐cycle arrest 53, cells do not stop growing *in vitro*, which might again involve an alternative survival strategy that can partially be explained by AHR activation. Microarray data for several human tumors, among them ES, revealed a correlation of *TDO2* expression with the expression of *CYP1B1* 14, suggesting that some ES tumors express *TDO2*, which might have therapeutic potential.

IL‐8, a pro‐inflammatory cytokine, is another AHR‐activated target gene 54 and mRNA strikingly increases in EFL cells, but can be down‐regulated by silencing *TDO2* (Fig. 4D). IL‐8 is involved in processes such as chemotaxis of target cells to the site of inflammation, stimulation of phagocytosis, and release of TNF‐related apoptosis‐inducing ligand (TRAIL) 55. Depending on the combination of stimuli, IL‐8 might also exert anti‐inflammatory signals 54. Intriguingly, in a concordant cytokine array, IL‐8 and IL‐6 were identified as up‐regulated upon low expression of EF, and functional analysis showed IL‐6‐mediated phosphorylation of signal transducer and activator of transcription 3 (STAT3) 56. Phosphorylated activated STAT3 is associated with tumor progression via favoring cell survival and proliferation 57. IL‐6 is one of AHR's target genes 14 and we can now speculate that ligand‐activated AHR functions as a mediator in this pathway.

To a certain extent, protumorigenic properties of TDO2 are mediated via the microenvironmental accumulation of breakdown products like KYN, KYNA, and their binding to AHR 58. The endogenous levels of KYN and KYNA in A673sh cells are in a high nanomolar to micromolar range which is in concordance with what has been reported to be sufficient for AHR activation 14, 59, and results in AHR translocation to the nucleus and activation. *In vivo* oncogenic potential of constitutively active AHR was described for hepatocarcinoma 60 and stomach tumor 61 progression, and *in vitro* the AHR has been reported to be overexpressed in several cancers, including lung carcinoma, gastric carcinoma, and medulloblastoma 62. In general, there is strong evidence of AHR activation being involved in tumor initiation, promotion and progression. Although AHR was discovered for its implication in detoxification, it is also involved in the activation of pro‐carcinogens causing DNA adduct formation 63 and favoring ligand‐mediated cell‐cycle progression 64. Intriguingly, AHR even mediates anti‐apoptotic effects, but only in a ligand‐dependent manner, as shown in *Myc* transgenic mice 65, in the promotion of ovarian tumors in rats 66, and cultured cell lines 67. However, AHR activation can stimulate anti‐ or protumorigenic pathways dependent on the cellular background or tumor context 68, 69. In general, ligand‐activated AHR is supposedly involved in carcinogenesis and tumor development, whereas the sole expression of AHR is mostly interconnected with low tumor burden or tumor suppression 21.

In summary, this is the first report to show ligand‐stimulated AHR activation in A673sh cells, caused by TDO2 activity and TRP breakdown products KYN and KYNA. In cells with low EF expression, this metabolic pathway can be switched on and might represent a novel alternative route of tumor cell survival, at least in the specific case of A673sh cells. It should be noted, however, that out of five ES cell lines studied by us, only A673sh cells induced *TDO2* after EF knockdown, while *IDO1* was consistently induced by IFNγ. However, analysis of primary human ES mRNA expression data sets reveal considerable variation in *TDO2* levels, correlated with *CYP1B1* as a sign of putative AHR activation. Thus, our finding, so far restricted to A673sh cells, may be of relevance to a subset of primary ES and/or subsets of primary tumor cells within a given tumor. Since the development of small molecule inhibitors against TDO2 70, targeting strategies have been discussed for many types of cancers where active TRP catabolism maintains the immunosuppressive environment 58. In the future, closer investigation of the tumor microenvironment from TDO2‐positive ES tumors could shed light onto metabolic alterations, potential AHR activation and, thus, help to identify those patients who might potentially profit from TDO2 directed therapy.

## Author contributions

CM and HK designed the study. CM and DF performed most of the experiments. RS, MK, AMK, FK, DNTA, SE, MG contributed to data generation. JA generated the inducible model cell line used in the study. CM and HK wrote the manuscript. All authors reviewed the manuscript.

## Supporting information


**Fig. S1.** IDO1 protein expression in A673sh cells.
