# Supplementary material for: EWS‐FLI1 impairs aryl hydrocarbon receptor activation by blocking tryptophan breakdown via the kynurenine pathway
Source: FEBS Lett. 2016 Jun 21;590(14):2063–75. doi: 10.1002/1873-3468.12243 (PMC4988508; doi:10.1002/1873-3468.12243)
Supplement: Supplementary file 1 — Fig. S1. IDO1 protein expression in A673sh cells. [file FEB2-590-2063-s001.pdf]

SUPPLEMENTARY MATERIAL

Figure S1

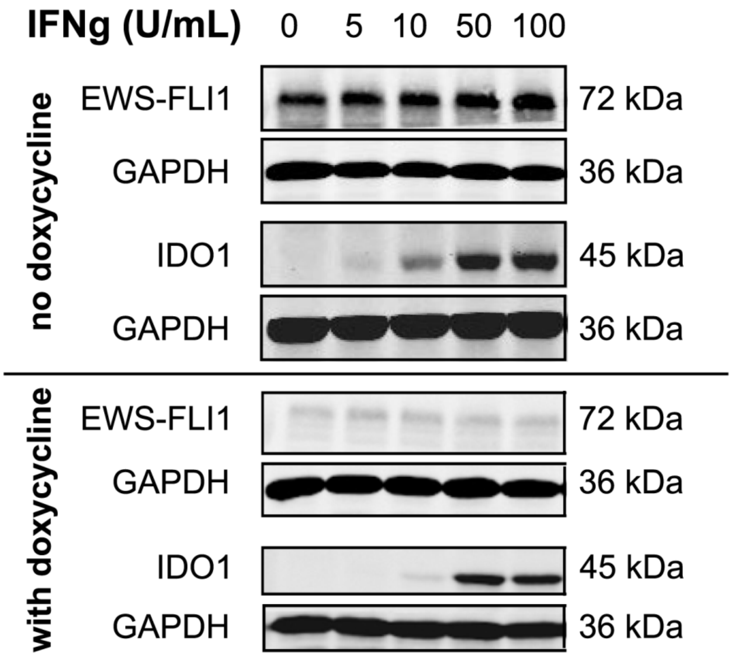

Figure S1: IDO1 protein expression in A673sh cells.

A673sh cells were treated with or without doxycycline (48 h) and treated with different concentrations of IFNγ from 5 – 100 U/mL for 24 hours (48 h doxycycline respectively). Protein was harvested and total protein was resolved by SDS-PAGE and immunoblotted with EWS-FLI1, IDO1, and GAPDH (loading control) antibodies. Upper panel: Western blot for cells without doxycycline treatment; lower panel: Western blot for A673sh cells in which EWS-FLI1 was silenced with doxycycline. These blots are representatives of two independent experiments. IFNγ was purchased from Preprotech, Rocky Hill, NJ, USA.
